# Updating the WHO Model Lists of Essential Medicines to promote global access to the most cost-effective and safe medicines for mental disorders

**DOI:** 10.1192/j.eurpsy.2024.182

**Published:** 2024-08-27

**Authors:** D. Papola, G. Ostuzzi, C. Gastaldon, C. Barbui

**Affiliations:** ^1^Neuroscience, Biomedicine and Movement Sciences, University of Verona, Verona, Italy; ^2^Global health and Social Medicine, Harvard Medical School, Boston, Massachusetts, United States

## Abstract

**Introduction:**

Since its first publication in 1977, the World Health Organization’s (WHO) Model List of Essential Medicines (EML) has guided the national procurement of medicines deemed essential to inform public health policy worldwide. Aiming to include the most effective, safe, and cost-effective medicines for priority conditions, WHO updates the EML every two years. However, over the past 45 years, updates to the mental health section of the EML have been infrequent, mostly involving the addition of individual medicines. A comprehensive revision of the entire section was never attempted.

**Objectives:**

The aim of this project was to update the mental health section of the EML to identify the most effective and safest medicines for mental disorders in the light of the most up-to-date evidence base.

**Methods:**

A series of nine evidence-based applications were submitted to the WHO Expert Committee on the Selection and Use of Essential Medicines in December 2022, recommending a substantial revision of the entire mental health section.

**Results:**

All of our applications were accepted by the WHO Expert Committee. For psychotic disorders, aripiprazole, olanzapine, paliperidone, and quetiapine were added as therapeutic alternatives to risperidone; short-acting intramuscular chlorpromazine was replaced by short-acting intramuscular olanzapine; first-generation antipsychotics were limited to oral haloperidol and chlorpromazine. For bipolar disorder, the list now includes second-generation antipsychotics such as quetiapine, aripiprazole, olanzapine, and paliperidone. Tricyclic antidepressants for depressive disorders were limited to amitriptyline alone. Treatment options for anxiety and obsessive-compulsive disorder are now expanded to include SSRIs. For anxiety disorders, diazepam and lorazepam became the only benzodiazepines recommended, with the specific caveat that they should only be used for short-term emergency treatment of acute and severe anxiety symptoms. Finally, chlorpromazine and haloperidol are no longer considered essential medications for psychotic disorders in children under 13 years of age.

**Conclusions:**

The WHO released the 23^rd^ EML in July 2023. After decades of minimal and inconsistent updates, groundbreaking changes have been made to its mental health section. The updated mental health section provides a compelling opportunity to improve the quality of medicine selection at the country level, with the goal of increasing the availability of the safest and most effective psychotropic medicines worldwide.

**Disclosure of Interest:**

None Declared

